# Construction of Stable Reporter Flaviviruses and Their Applications

**DOI:** 10.3390/v12101082

**Published:** 2020-09-25

**Authors:** Coleman Baker, Pei-Yong Shi

**Affiliations:** 1 Microbiology and Immunology Department, University of Texas Medical Branch, Galveston, TX 77555, USA; ckbaker@utmb.edu; 2 Biochemistry and Molecular Biology Department, University of Texas Medical Branch, Galveston, TX 77555, USA; 3 Institute for Human Infections & Immunity, University of Texas Medical Branch, Galveston, TX 77555, USA; 4 Institute for Translational Science, University of Texas Medical Branch, Galveston, TX 77555, USA; 5 Sealy Institute for Vaccine Sciences, University of Texas Medical Branch, Galveston, TX 77555, USA; 6 Sealy Center for Structural Biology & Molecular Biophysics, University of Texas Medical Branch, Galveston, TX 77555, USA

**Keywords:** reporter viruses, RNA viruses, RNA, emerging viruses, zoonosis

## Abstract

Flaviviruses are significant human pathogens that cause frequent emerging and reemerging epidemics around the world. Better molecular tools for studying, diagnosing, and treating these diseases are needed. Reporter viruses represent potent tools to fill this gap but have been hindered by genetic instability. Recent advances have overcome these hurdles, opening the way for increased use of stable reporter flaviviruses to diagnose infections, screen and study antiviral compounds, and serve as potential vaccine vectors.

## 1. Introduction

The past two decades have been a lesson in the potential of unknown or unrecognized RNA viruses to emerge from animal reservoirs in new locations to cause local epidemics or widespread pandemics. In 1999, the United States saw the introduction of West Nile virus, which has caused tens of thousands of cases of neurological disease [1]. Subsequently, 2002 saw the first outbreak caused by a new coronavirus, severe acute respiratory syndrome coronavirus (SARS CoV), which infected thousands of people before control measures ended its spread [2]. The yearly influenza turned particularly worrisome in 2009 with the emergence of an especially deadly strain of influenza A with swine origins that killed an estimated 284,000 people worldwide [3]. Notably, the second novel coronavirus outbreak happened in 2012, with the causative agent, middle eastern respiratory syndrome coronavirus (MERS CoV), causing over six hundred deaths [2]. The largest Ebola virus outbreak ravaged West Africa for four years, starting in 2013 [4]. Shortly thereafter, the little-known mosquito-borne Zika virus shocked scientists after its introduction to the Americas demonstrated its non-vectored transmission and ability to cause fetal malformations [5]. Finally, the current pandemic of coronavirus infectious disease 2019 (COVID-19), caused by yet another novel coronavirus, continues to spread worldwide, with cases currently over 30 million and 953,072 deaths globally [6]. Each of these instances has demonstrated the progress still to be made in studying and counteracting these and future diseases.

Two of the above examples from the past twenty years, West Nile (WNV) and Zika (ZIKV) viruses, are part of the insect-vectored pathogens from the Flavivirus family. This family also includes the mosquito-transmitted dengue virus (DENV), Japanese encephalitis virus (JEV), and yellow fever virus (YFV), as well as the tick-transmitted tick-borne encephalitis virus (TBEV), Langat virus (LGTV), and Powassan virus (POWV). Collectively, these viruses cause greater than 390 million infections yearly, with the majority of those being from the four serotypes of dengue virus [7]. Along with the more well-known and studied of the Flaviviruses, come a number of lesser known but emerging family members with outbreak potential [1]. Discouragingly, current medical countermeasures are restricted to vaccines with limited availability or efficacy and no direct-acting antivirals [8]. Tools and resources to bridge these gaps are needed in order to prepare for the next flavivirus outbreak. Reporter viruses, or viruses that carry an engineered gene to allow for easier visualization or quantification, have an immense potential to fill these voids and accelerate the development of flavivirus diagnostics, antivirals, and vaccines.

**Figure 1:**
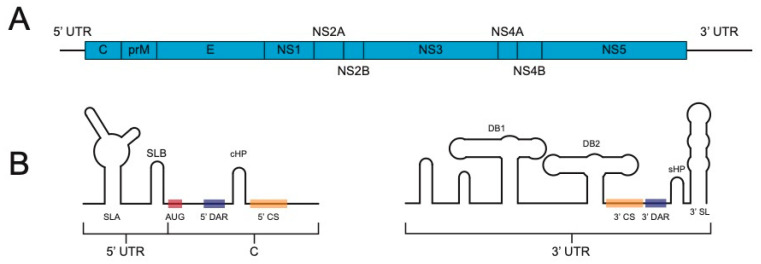
Flavivirus genome. (**A**). General flavivirus genome organization. C, capsid; prM, pre-membrane; E, envelope; NS, nonstructural. (**B**). Depiction of some of the important RNA structures and sequences at the 5′ and 3′ ends of the flavivirus genome. SLA, stem loop A; SLB, stem loop B; AUG, start codon; 5′ DAR, 5′ downstream of AUG region; cHP, conserved hairpin; 5′ CS, 5′ cyclization sequence; DB1, dumbbell 1; DB2, dumbbell 2; 3′ CS, 3′ cyclization sequence; 3′ DAR, 3′ downstream of AUG sequence. Note that some of the structures and sequences at the 5′ end are within the coding region of the capsid gene.

The Flavivirus family consists of positive sense, single-stranded RNA viruses. Their ~11 kb genome is translated as one large polyprotein that is co- and post-translationally cleaved into three structural proteins (capsid [C], pre-membrane [prM], and envelope [E]) and seven non-structural proteins (NS1, NS2A, NS2B, NS3, NS4A, NS4B, and NS5) by cellular and viral proteases (Figure 1A).

The genome’s single open reading frame is flanked, on both the 5′ and 3′ ends, by an untranslated region (UTR) that, among other functions, contain signals, such as a cyclization sequence, that are critical for RNA replication (reviewed in [9], Figure 1B). The cloning and subsequent manipulation and experimentation of flavivirus genomes has resulted in a greater understanding of the viral and host determinants of infection in both mammalian and insect hosts [10] as well as paved the way for designed vaccines [11,12] and tools, such as replicons [13] and reporter viruses. Reporter viruses enable simple, rapid, and high-throughput quantification of viruses in a variety of settings and applications, making them powerful tools for research, diagnosis, and medicine. 

In this review, we summarize and discuss the development and shortcomings of reporter flaviviruses, covering different methods of construction and their influence on the long-standing problem of genetic instability. We also cover multiple recent and novel methods that have been used to overcome the problem of instability. These are summarized by chronological order in Table 1. A discussion of the current applications of reporter flaviviruses is included, as well as how current and future applications can benefit from the increased stability afforded by recent advances.

## 2. Timeline of Reporter Flavivirus Design

The first reporter flavivirus constructs were made with replicon RNAs, which are autonomously replicating viral RNAs that lack the structural proteins necessary for viral particle formation. Replicons for Kunjin virus (KUNV) were first engineered with a chloramphenicol acetyltransferase (CAT) gene [14], followed by a green fluorescent protein (GFP) gene [15] in a permissible location in the 3′ UTR immediately following an internal ribosome entry site (IRES). Similar work was also done with WNV [42]. Replicons are powerful tools for viral replication experiments and testing drugs that affect viral replication, especially as they require less strict containment measures, but they lack viral structural proteins and do not complete a full viral life cycle. The KUNV replicon approach was later applied for the establishment of a hepatis C virus (HCV) replicon system [43,44] that was essential for the development of successful HCV therapeutics.

Full-length infectious viruses can be more difficult to clone due to instability in their bacterial plasmids, but they do not have the shortcomings of replicons, which do not cover virus entry/fusion and virion assembly/release. The bacterial instability challenge was first overcome by in vitro ligation of cDNA fragments that cover the complete YFV genomic RNA sequence [45]. Similar approaches were used to develop infectious clones for JEV [46], DENV2 [47], and the TBEV strain Hypr [48]. For other flaviviruses, stable full-length infectious clones were established for DENV-4 [49], KUNV [50], the TBEV strain Neudoerfl [48], Murray Valley encephalitis virus (MVEV) [51], the TBEV strain Langat [52], and the WNV epidemic strain [53]. The key solution to overcome the stability issue when amplifying cDNA clones in *Escherichia. coli* is to use low copy number plasmid vectors.

**Figure 2:**
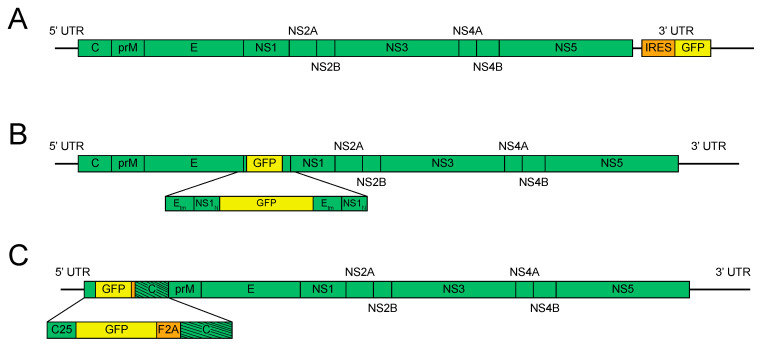
Flavivirus reporter schemes—less stable. (**A**). 3′ untranslated region (UTR) reporter insertion. The reporter gene is inserted into a permissive site in the 3′ UTR under the control of an internal ribosomal entry site (IRES). (**B**). E/NS1 reporter insertion. The reporter gene is placed at the junction between E and NS1, with a duplication of the N-terminus (N) of NS1 and the transmembrane (tm) domains of E. (**C**). 5′ reporter insertion. The reporter gene is placed at the junction of the 5′ UTR and the capsid gene. The first 25 amino acids of the capsid are duplicated (C25) and the reporter gene is followed by the foot and mouth disease virus 2A sequence (F2A) and codon scrambled capsid gene (represented by the slanted lines).

The first report of a full-length reporter flavivirus came in 2003 using JEV [16], which was engineered with GFP and luciferase in the 3′ UTR, similar to the previously published KUNV replicons (Figure 2A).

Successively, two reports of infectious reporter WNV were published in 2005, one with green fluorescent protein (GFP) [17], and one with Renilla luciferase (RLuc) [18]; both reporter genes were placed in the 3′ UTR under the control of an IRES. These reports both document the genetic instability problem that plagued reporter flaviviruses for the next 15 years. In the first report, genetic stability was assayed by serial passage in human embryonic kidney (HEK293T) cells followed by analyzing the cells by flow cytometry for E protein and GFP signals after 48 h of infection. Greater than 90% of the cells stained positive for E protein, while <60% were GFP positive at 48 and 96 h, and GFP positive cells dropped to <10% by 144 h. Viral RNA was also harvested at different times post-infection on baby hamster kidney (BHK-21) cells and used as a template for RT-PCR. The band size of the products was resolved on an agarose gel using electrophoresis, showing a decrease in the size of the amplicon containing GFP as time passed. Cloning and sequencing of these amplicons indicated that deletions across the IRES/GFP sequences were occurring, implicating recombination as the source of genetic instability [17]. This report also showed that placement of a reporter gene attenuated viral growth when compared to the parental virus. The second report, using WNV-RLuc, passaged the virus seven times on BHK cells, followed by RT-PCR on viral RNA. The RT-PCR product of the 3′ UTR matched the size of the product from WT WNV and not reporter WNV. These results corroborated those from WNV-GFP and established RT-PCR on passaged viral RNA as the traditional assay to assess genetic stability. Similar versions of other reporter flaviviruses, such as DENV [19,24] and ZIKV [38], using an IRES in the 3′ UTR have been made, though not every report details the instability of the reporter gene.

Another less-used strategy for constructing reporter flaviviruses was first published in 2007 [20]. In short, enhanced GFP (EGFP) was engineered in the 17D vaccine strain of YFV at the junction between viral envelope protein (E) and nonstructural protein 1 (NS1), with the stem–anchor domain of E and amino-terminus of NS1 duplicated to maintain correct proteolytic processing and membrane orientation (Figure 2B). Notably, duplicated regions of the genome can lead to increased levels of homology-directed recombination [54], which had already been associated with reporter virus instability. Despite this, the authors did not report codon scrambling of the duplicated sequences to decrease homologous recombination. Stability results from this construct were mixed, with two of five plaque-purified viral populations showing loss of reporter signal between passages five and ten in Vero cells by flow cytometry. This method has been used to design EGFP reporter LGTV [31], a tick-borne flavivirus, and Nanoluciferase (NanoLuc) and EGFP ZIKV constructs [39]. The E/NS1 NanoLuc ZIKV showed stability to ten passages but grew to lower titers than other reporter schemes. However, EGFP ZIKV did not have detectable fluorescence after transfection, leading the authors to conclude that this region is more restrictive in the sequences that can be engineered there. Our own experience affirms that this method is less robust than those described hereafter. Of note though, this strategy was used to create a YFV-expressing simian immunodeficiency virus (SIV) gag protein and, after modifying the gag sequence, it was reported stable up to twenty passages [27].

The most robust scheme for reporter flavivirus construction to date was published in 2007, using YFV [21]. In this method, EGFP was placed between the 5′ UTR and the capsid gene. Importantly, RNA signals for replication can be found in both the 5′ UTR and the capsid gene, so a portion of the capsid was necessarily duplicated upstream of the reporter gene. The reporter gene was then followed by the 2A sequence from foot and mouth disease virus (FMDV, F2A) to ensure EGFP separation from the polyprotein. To reduce homology and usage of the downstream 5′ cyclization sequence, the codon sequence of the complete capsid gene was optimized (Figure 2C). To assess genetic stability, the virus was passaged five times in BHK-21 cells. Cells were then stained for viral antigen and analyzed by flow cytometry for both viral antigen and GFP. The results indicated that 11% of the viruses had lost GFP signal, indicating this strategy to be more stable than IRES-driven cassettes in the 3′ UTR.

The capsid duplication strategy has been successfully applied to many other flaviviruses, with slight variations giving different results in stability and utility, though addition of a reporter gene causes general attenuation in all cases [22,23,25,26,28,29,30,32,33,35,36,39]. As a rule, reporter viruses made without reducing the homology between duplicated capsids show marked instability, similar to the 3′ UTR reporter viruses [23,29,30,33,35]. On the other hand, reducing the homology increases stability, reported usually out to five passages in cell culture [25,26,32,35]. Case in point, Reporter WNV and JEV with no homology reduction were initially shown to be unstable. Then, multiple rounds of plaque purification led to the selection of stable variants that all contained a mutation in the downstream copy of the 5′ cyclization sequence [29,33], lending evidence that decreasing homology, perhaps especially in the cyclization sequence, can increase stability. Engineering of similar mutations in other flaviviruses, on the other hand, has not led to similar levels of stability [26]. Lastly, a report using DENV indicated that doubling the 2A sequence immediately downstream of the reporter gene could increase genetic stability by up to two or three passages [35].

## 3. Methods for Further Stabilization of Reporter Flaviviruses

As seen throughout the decade following the first reporter flavivirus, better design methods have resulted in more stable and more robust reporter viruses. Despite these improvements, interest still remains in engineering increased stability. Viruses carrying reporter genes with long-standing stability can be used in long-term pathology experiments, such as experiments involving transmission among multiple hosts, in diagnostic applications where reliability of the reporter signal is crucial, and in industrial applications where large viral batches are needed from small seed stocks. Therefore, recent work has shown multiple strategies that can be applied to ensure engineered reporter genes are maintained.

**Figure 3:**
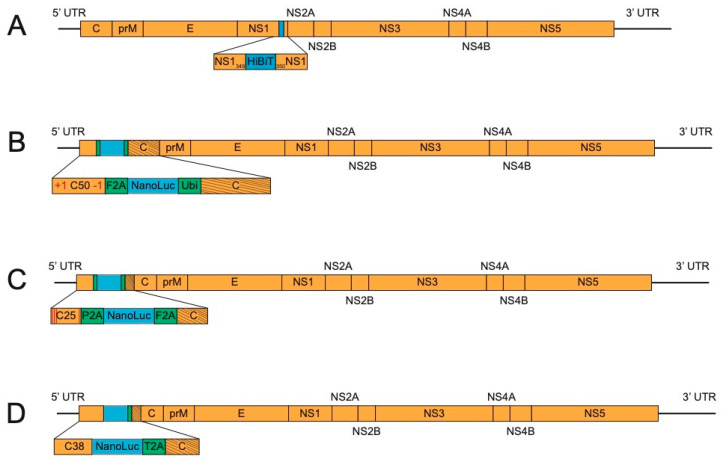
Flavivirus reporter schemes—stable. (**A**). NS1 insertion of split luciferase. The small subunit of split NanoLuc (HiBit) is inserted in NS1 at amino acid 349. (**B**). C50 with frameshift mutation. The reporter gene is engineered after a duplication of 50 capsid amino acids (C50) and flanked by F2A and the ubiquitin sequence (Ubi). C50 contains a +1-frameshift mutation after the fourth codon, which is restored at the end of C50. Slanted lines indicate codon scrambling. (**C**). Recombination-dependent lethal mutations. The reporter gene is inserted at the 5’ end of the genome after a 25 amino acid capsid duplication (C25) and flanked by different 2A sequences (F2A and porcine teschovirus-1 2A, P2A). The C25 region contains four amino acid mutations, denoted by red lines. Slanted lines in the capsid gene represent codon scrambled sequence. (**D**). Lengthened capsid duplication. The reporter gene is located at the beginning of the capsid gene, with a duplication of 38 amino acids. Slanted lines in the capsid gene correspond to the 38 codons that have been scrambled (T2A, thosea asigna virus 2A).

Interest in split reporter proteins, where the large subunit of the protein becomes active after addition of a smaller, critical subunit, has recently increased because of their minimal genome perturbation and ability to be introduced by CRISPR technology [55]. Such a system has been developed for NanoLuc [56] and was applied to JEV and DENV4 by Tamura et al. [34]. The small size of the insert, 57 nucleotides, was initially engineered after the E protein and includes a linker sequence, the small NanoLuc subunit (HiBit), and a small NS1 duplication to ensure proper polyprotein processing. Stability was initially shown out to five passages and, notably, reporter viruses replicated similarly to parental viruses in vitro. Later, this work was further expanded with experiments in mice infected with JEV-HiBit [37]. Reporter virus-infected mice showed less mortality than those infected by WT JEV, highlighting the attenuation that even a small insert can cause. Further optimization of the insertion site after protein modeling, in vitro screening, and in vivo experiments led to a JEV-HiBit virus with an insert at NS1_349_, with no duplication of NS1 (Figure 3A).

This improved reporter virus was stable to ten passages in Huh7 cells and, remarkably, led to similar mortality as WT JEV in immunocompetent mice. This leads to a potent tool for assessing viral titers in in vitro and in vivo experiments, with the drawback of being unable to do live animal imaging because of the challenges of supplying the large NanoLuc subunit and the secretory nature of NS1.

Volkova et al. developed a separate, elegant technique using ZIKV bearing NanoLuc as a model [39]. They built on the capsid duplication method, noting that, for ZIKV, the reported sizes used for the duplication ranged from 25 amino acids [28], to 33 amino acids [30], and even duplication of the full capsid [32]. They methodically tested duplication lengths, concluding that a duplication of 50 amino acids (C50) is the shortest length that replicates similarly to WT ZIKV. To further ensure stability, a +1-frameshift mutation was introduced at the beginning of the C50 and restored at the end of the C50 (Figure 3B). This strategy preserves the RNA elements in the C50 but changes the amino acids that are translated. If recombination deletes the engineered NanoLuc, the frameshift mutation is introduced into the polyprotein, resulting in mistranslation. This method secured the NanoLuc gene for ten passages.

Concurrently, we developed a similar technique as Volkova et al. for long-term stabilization of NanoLuc ZIKV and YFV [40]. We also realized that if the protein sequence of the duplicated capsid (C25) could be disrupted without disturbing the critical RNA elements, recombined viruses could be hindered. Such recombination-dependent lethal mutations were designed based on the work of Samsa et al. [23], which showed that DENV requires a threshold of positively charged amino acids in the N-terminus of the capsid protein. Thus, charge-reversing mutations were designed in the C25 of ZIKV and YFV (Figure 3C). When recombination occurred, these mutations were brought into the full capsid and prevented it from functioning in viral particle formation. As a result, these mutations extended the stability of NanoLuc ZIKV and YFV from P5 to greater than P10. These viruses were then used in rapid reporter-based neutralization assays.

In working to bring this technique to other relevant flaviviruses, we established a panel of NanoLuc viruses, including ZIKV, YFV, DENV1-4, JEV, and WNV. In passaging these viruses, we noted that those with longer capsid duplications (DENV1-4 (C38), JEV (C34), and WNV (C33)) were stable to ten passages, while those with C25 (ZIKV and YFV), were only stable to five passages. We hypothesized that an extended capsid length, in itself, would be enough to stabilize reporter flaviviruses when codon homology was scrambled. This could be due to RNA signals present beyond C25 that may not be mandatory, but still assist in genome replication. Indeed, ZIKV and YFV with 38 amino acid capsid duplications (Figure 3D) were made and found to be stable out to ten passages in cell culture [41]. These results indicate that optimization of replication conditions may be all that is necessary to increase genetic stability.

## 4. Applications of Reporter Flaviviruses-Present and Future

Reporter genes, including fluorescent and bioluminescent genes, allow for easier quantification of virus levels. This simple concept has seen many applications that advance virus and disease understanding, as well as countermeasure development. One of the earliest reports of reporter flaviviruses was in conjunction with antiviral compound testing. Traditionally, these tests are done by adding virus and putative antiviral compounds to relevant cells. Following multiple rounds of viral replication, supernatant samples are assayed by plaque- or focus-forming assay, which take up to five days or more to obtain results and are not amenable to high-throughput. Using reporter viruses, results can be obtained using reporter output in place of an infectious virus, increasing throughput, turnaround, and ease of quantification [18,24,25,28,29,40,57]. This technology has enabled high-throughput compound library screens, in which hundreds to thousands of potential antiviral compounds can be tested.

Similar to antiviral testing, conventional testing for neutralizing antibodies by plaque reduction neutralization tests (PRNT) is a long and labor-intensive process. Several methods for using reporter gene output, instead of viral titers, have been described to shorten this assay from a week to as little as 4 h and increase throughput by using either a flow cytometer or a plate reader [40,58,59,60]. In addition, the ability to make chimeric reporter flaviviruses, or viruses with heterologous prM-E genes, has the potential to quickly expand this method to many other flaviviruses [61,62,63]. However, it remains to be determined if a chimeric virus containing a heterologous viral prM-E could be equally neutralized by positive serum specimens as an authentic virus without chimeric prM-E. Neutralization assays play an important role in both vaccine efficacy trials and flavivirus diagnostics, and, as such, stable reporter constructs have great potential to hasten both of these pursuits. In both cases, these laboratories are not routinely equipped with necessary equipment to rescue stock reporter viruses when more are required. Stable reporter viruses allow these crucial laboratories to amplify viral stocks many times over and maintain confidence in the reporter signal output. Thus, recent advances could allow this technology to be used more easily and routinely, increasing the pace of disease diagnosis and vaccine trials.

Another powerful application of reporter flaviviruses comes in the use of luciferase-carrying viruses to be quantified in living animals. Carefully engineered cameras can detect luciferase signals in infected mice, allowing for temporal quantification and tracking of infection in the same mice. This real-time view of infection, known as bioluminescent imaging (BLI), also reduces the number of mice needed and can represent viable models for both antiviral and vaccine studies. Such studies have already been done with DENV2 [26] and JEV [33]. More stable reporter viruses could be used in a similar fashion to model not only pathology or study medical countermeasures, but also host–host transmission over an extended time. As the flavivirus life cycle involves both insect and mammalian hosts, this type of proposed model could benefit our understanding of this complex progression.

The possibility of approved flavivirus vaccine strains, specifically the 17D strain of YFV, being used to deliver heterologous antigens as a vaccine has long been considered and studied. YF17D was initially restricted to small T and B cell epitopes engineered either in a space in the envelope protein [64,65] or between NS2B and NS3 [66,67,68] and was, thus, limited in scope and efficacy. Longer antigens have been developed in the E/NS1 junction (see Figure 2B) with mixed results [27,69]. The newly developed gene stabilization methods for flaviviruses open the door to reliable delivery of larger heterologous antigens that can be used as experimental vaccines. As YF17D is one of the oldest and best performing vaccines available, this method represents a promising pathway for rapid vaccine development against new and emerging pathogens. 

## 5. Conclusions

Since first creation, reporter flaviviruses have found uses in molecular virology [22,23], antiviral drug studies [18,24,25], pathology studies (including live animal imaging) [26,33,37], and neutralizing antibody quantification [40,41,58,59]. Initial methods of reporter virus construction, placing an IRES driven gene in the 3′ UTR, were found to be genetically unstable due to recombination. Subsequent methods using the 5′ UTR-capsid junction proved more robust and stable if homology was reduced in duplicated capsid regions. Further improvements to achieve long-term stability, usually regarded as stable for ten passages in cell culture, have also been developed. Mutations to shift the reading frame or change the protein charge in the duplicated capsid can be used to block recombined viruses and extend stability. Additionally, optimizations of capsid duplication length can also increase reporter stability, as can using split reporter systems. These new methods have opened further doors for reporter flaviviruses, including their broad use in flavivirus diagnostics, long-term pathological experiments involving multi-host transmission, and possible antigen delivery as experimental vaccines for the current or future pandemic. To further improve the reporter flavivirus platform, future studies are needed to address two weaknesses of the current systems. First, the insertion of a reporter gene attenuates the engineered flavivirus. Such attenuation weakens the utility of the current reporter viruses when studying viral replication, pathogenesis, and transmission between vertebrate and insect hosts. Second, stable reporter flaviviruses still only allow insertion of relatively small foreign genes. Larger genes cannot be stably accommodated by flaviviruses, possibly due to packaging limitations imposed by viral RNA genome size. To increase the application as a viral delivery vehicle, it is important to further improve the vector to enable the delivery of larger cargo.

## Figures and Tables

**Table 1:** Summary of reporter flaviviruses.

Year of Publication	Reporter Gene and Flavivirus	Reference
**1997**	CAT KUNV replicons	[14]
**1999**	GFP KUNV replicon	[15]
**2003**	IRES-GFP/Luc JEV	[16]
**2005**	IRES-GFP WNV	[17]
**2005**	IRES-RLuc WNV	[18]
**2007**	IRES-RLuc DENV2	[19]
**2007**	E/NS1-GFP YFV	[20]
**2007**	5′-GFP YFV	[21]
**2009**	5′-RLuc DENV2	[22,23]
**2010**	IRES-RLuc DENV2	[24]
**2011**	5′-RLuc DENV2	[25]
**2012**	5′-GFP/FLuc DENV2	[26]
**2014**	E/NS1-gag YFV	[27]
**2016**	5′-RLuc ZIKV	[28]
**2016**	5′-GLuc WNV	[29]
**2016**	5′-GFP ZIKV	[30]
**2017**	E/NS1-GFP LGTV	[31]
**2017**	5′-GFP/mCherry/NanoLuc ZIKV	[32]
**2017**	5′-RLuc JEV	[33]
**2017**	NS1-HiBiT JEV/DENV4	[34]
**2018**	5′-GFP/Clover2/bfloGFP DENV2	[35]
**2019**	5′ NanoLuc DTMUV	[36]
**2019**	NS1-HiBiT JEV	[37]
**2020**	IRES-NanoLuc/GFP ZIKV	[38]
**2020**	E/NS1-NanoLuc/GFP ZIKV	[39]
**2020**	5′-GFP/NanoLuc ZIKV	[39]
**2020**	5′ w/+1C-1 NanoLuc/GFP ZIKV	[39]
**2020**	5′ w/ΔC-NanoLuc ZIKV/YFV	[40]
**2020**	5′ C33-38 NanoLuc ZIKV/YFV/DENV1-4/JEV/WNV	[41]
